# In Vitro Inhibition of Zika Virus Replication with Poly(Sodium 4-Styrenesulfonate)

**DOI:** 10.3390/v12090926

**Published:** 2020-08-23

**Authors:** Paweł Botwina, Magdalena Obłoza, Artur Szczepański, Krzysztof Szczubiałka, Maria Nowakowska, Krzysztof Pyrć

**Affiliations:** 1 Virogenetics Laboratory of Virology, Malopolska Centre of Biotechnology, Jagiellonian University, Gronostajowa 7a, 30-387 Krakow, Poland; pawel.botwina@doctoral.uj.edu.pl (P.B.); artur.szczepanski@doctoral.uj.edu.pl (A.S.); 2 Department of Microbiology, Faculty of Biochemistry, Biophysics and Biotechnology, Jagiellonian University, Gronostajowa 7, 30-387 Krakow, Poland; 3 Faculty of Chemistry, Jagiellonian University, Gronostajowa 2, 30-387 Krakow, Poland; m.obloza@uj.edu.pl (M.O.); szczubia@chemia.uj.edu.pl (K.S.); nowakows@chemia.uj.edu.pl (M.N.)

**Keywords:** Zika virus, antiviral, polymer, cell culture, flavivirus

## Abstract

Zika virus (ZIKV) is an emerging mosquito-borne pathogen associated with microcephaly and other congenital abnormalities in newborns as well as neurologic complications in adults. The explosive transmission of the virus in the last ten years put it in the limelight and improved our understanding of its biology and pathology. Currently, no vaccine or drugs are available to prevent or treat ZIKV infections. Knowing the potential of flaviviruses to broaden their geographic distribution, as observed for the West Nile virus, it is of importance to develop novel antiviral strategies. In this work, we identified poly(sodium 4-styrenesulfonate) (PSSNa) as a new polymeric ZIKV inhibitor. We demonstrated that PSSNa inhibits ZIKV replication in vitro both in animal and human cells, while no cytotoxicity is observed. Our mechanistic studies indicated that PSSNa acts mostly through direct binding to ZIKV particle and blocking its attachment to the host cells.

## 1. Introduction

Zika virus (ZIKV) belongs to the Flaviviridae family, which encompasses viruses such as dengue virus, yellow fever virus, West Nile virus, tick-borne encephalitis virus, and Japanese encephalitis virus [1]. The ZIKV is a mosquito-borne arbovirus, for which Aedes mosquitoes are competent vectors. The virus is transmitted mainly by Aedes aegypti, known to be active during the morning hours, but other Aedes species, including Aedes albopictus, has also been reported. The Aedes mosquitoes are amongst the invasive species, expanding its geographic range which consequently brings the threat of arboviral infections also to the temperate zones [2]. Until now, ZIKV infections have been confirmed in 91 countries in South and Central America, Central Africa, South Asia, the South Pacific region, and Europe [3,4].

The infection is frequently asymptomatic (80% of cases), and the disease associated with the virus is mild-to-moderate [5]. In symptomatic patients, it manifests itself with flu-like symptoms and in some cases is associated with rash and itching [6]. For that reason, the disease was neglected for years, and it was not considered to be a healthcare threat. After the epidemic spread in 2016, however, it was noted that, in some cases, it had severe sequelae. The most striking effect is observed in pregnant women if the mother is infected with ZIKV during the first trimester of pregnancy, as congenital malformations of the fetus occur in about 11% of cases [7,8]. The symptoms include increased muscle tone, hyperreflexia, spasticity, paroxysmal cramps, brain calcification, brain atrophy, and enlarged brain chambers [9]. No defects have been observed in neonates born to mothers infected in the second or third trimester of pregnancy [10]. Less severe, but also notable consequences of the infection were observed in adults and included Guillain–Barré syndrome and other neurological disorders. Up to four weeks after the infection, neurological symptoms may occur in the form of acute inflammatory demyelinating polyneuropathy, and approximately 50% of patients with neurologic sequelae experience bilateral facial palsy [11,12]. The pathomechanism leading to this syndrome is not yet fully understood. It is suspected that it may be related to the direct effect of the virus on nerve cells or excessive immune response to infection. Until now, no vaccine or drug is available to prevent or treat ZIKV infection.

Polymeric compounds, both natural and synthetic, exhibit a broad spectrum of antibacterial and antiviral properties. Their activity is often associated with their ionic charge, which can directly interact with the biological structures of both viruses and cells via electrostatic interactions, inhibiting the process of adhesion and/or entry of the virus into the host cell [13].

We and others found highly charged polymeric compounds as effective replication inhibitors of human and animal viruses. These include natural and synthetic polymers and their derivatives. Both cationic and anionic macromolecules were shown to be effective, including carrageenans, heparan sulfate, and sulfate derivatives of cellulose and lignin, dextran, chitosan, curdlan, xylan, and fucoidans and synthetic polymers such as sulfonated derivatives of poly(allylamine hydrochloride) (NSPAHs), dendrimers containing sulfonic groups, poly(sodium styrenesulfonate) and its copolymer with maleic acid, poly(methacrylic acid) functionalized with ribavirin, telomerized ω-acryloyl anionic surfactants and poly(ethylene glycol)-block-poly(3-(methacryloylamino)propyl trimethylammonium chloride) (PEG-b-PMAPTAC). These and many other polymers hamper a variety of pathogenic viruses, such as influenza A and B virus [14,15], herpesviruses [16,17,18,19,20,21,22,23,24,25], human immunodeficiency virus (HIV) [23,26,27], human metapneumovirus (hMPV) [28], and other viruses in in vitro and ex vivo models [29,30,31,32,33,34]. In vitro studies have shown that highly sulfated heparin, dextran sulfate, and suramin significantly inhibit ZIKV infection in Vero cells [35].

The current work aimed to examine the inhibitory properties of poly(sodium 4-styrenesulfonate) (PSSNa) on the ZIKV replication in vitro. Antiviral properties of PSSNa have already been described for both human and animal pathogenic viruses [21,22]. Here, we show very potent inhibition of ZIKV by PSSNa in different cell systems. Studies on the mechanism of action revealed that PSSNa interacts directly with ZIKV, preventing its attachment to the host cell. Showing low cytotoxicity towards different cells, very potent anti-ZIKV properties, and beneficial physicochemical properties, PSSNa is a promising drug candidate for treating ZIKV infections.

## 2. Materials and Methods

### 2.1. Polymeric Inhibitors

A series of well-defined, high purity (analytical standards), poly(sodium 4-styrenesulfonate) (PSSNa) samples of the average molecular weight (MW) in the range of 1–3160 kDa and the low dispersity (Mw/Mn = 1.05–1.20) were purchased from Perfect Separation Solutions (PSS) Polymer Standards Service (Amherst, MA, USA). Polymeric stock solutions were prepared in distilled water supplemented with penicillin (100 U/mL) and streptomycin (100 µg/mL) and stored at 4 °C.

### 2.2. Cells and Virus

U251 (Human Glioblastoma), Vero (Cercopithecus aethiops kidney epithelial, ATCC CCL-81), and primary human skin fibroblast (HSF) cells were maintained under Dulbecco-modified Eagle’s medium (DMEM; high glucose, Life Technologies, Eugene, OR, USA) supplemented with 10% heat-inactivated fetal bovine serum (FBS; Life Technologies, Eugene, OR, USA), penicillin (100 U/mL), and streptomycin (100 μg/mL) at 37 °C in an atmosphere containing 5% CO_2_.

Zika virus strains, H/PF/2013, MR-776, PRAVABC59, Human/2015/Honduras and Mosquito/1966/Malaysia, were acquired from BEI Resources (Manassas, VA, USA). Stocks of ZIKV were obtained by infecting 90% confluent Vero cells with TCID50 = 400/mL. The cytopathic effect (CPE) was observed 3–4 days post-infection. Cells were lyzed by three consecutive freeze–thaw cycles, and the supernatant was collected, aliquoted, and stored at −80 °C. The TCID50 value of the stock was measured using Reed and Muench method [36]. The mock sample was prepared in parallel using non infected cells.

### 2.3. Cell Viability

XTT Cell Viability Assay kit (Biological Industries Cromwell, CT, USA) was used according to the manufacturer’s instructions. Briefly, cells were incubated with PSSNa at different concentrations of for three (Vero cells), four (U251), or seven days (HSF cells) at 37 °C. After incubation, the medium was discarded, and 100 μL of fresh medium was overlaid on cells. Then, 50 μL of the activated 2,3-bis-(2-methoxy-4-nitro-5-sulphenyl)-(2H)-tetrazolium-5-carboxanilide (XTT) solution was added, and samples were incubated for 2 h at 37 °C. The absorbance (λ = 450 nm) was measured using Spectra MAX 250 spectrophotometer (Molecular Devices, San Jose, CA, USA). Data were presented as the ratio of signal from the tested sample and the control sample (solvent-treated cells) × 100%.

### 2.4. RNA Isolation and RT-qPCR

Isolation of viral RNA was carried out using a commercially available RNA isolation kit (Viral DNA/RNA Isolation Kit, A&A Biotechnology, Poland) according to the protocol provided by the manufacturer. Isolated RNA was subjected to reverse transcription (RT) and quantitative real-time PCR (RT-qPCR) using the GoTaq^®^ Probe 1-Step RT-qPCR System Protocol kit (Promega, Madison, WI, USA). Due to the well-known ability of highly charged polymers to affect the RNA isolation process, the supernatants were diluted 1000-fold prior to isolation [28].

The RT-qPCR reaction was carried out using 3 μL of isolated viral RNA, which was reverse transcribed and amplified in a 10 μL reaction containing 1 × GoScript ™ RT Mix for 1-Step RT-qPCR, 1× GoTaq Probe qPCR Master Mix with dUTP, 300 nM specific probe labeled with 6-carboxyfluorescein (FAM) and 6-carboxytetramethylrhodamine (TAMRA) (5′ FAM–CGG CAT ACA GCA TCA GGT GCA TAG GAG-TAMRA-3′), and 450 nM of each primer (5′ TTG GTC ATG ATA CTG CTG ATT GC 3′ and 5′ CCT TCC ACA AAG TCC CTA TTG C 3′). The reaction was carried out in a thermal cycler (CFX96 Touch Real-197 Time PCR Detection System, Bio-Rad) under the following conditions: 45 °C 15 min (reverse transcription), 95 °C 2 min, then 40 cycles of 15 s at 95 °C and 30 s at 60 °C. Appropriate standards were prepared to evaluate the number of viral RNA molecules in the sample.

### 2.5. Mechanism of Action Assays

To determine at which step PSSNa hampers the ZIKV replication, a set of mechanistic assays was performed as described before [37].

Assay I, “Virus inactivation assay”, verified the direct inactivation of the virus by the tested compound. Virions (TCID50 = 400/mL) were incubated with PSSNa (500 µg/mL) for 30 min at room temperature with mixing. After preincubation, samples were titrated on confluent cells.

Assay II, “Cell protection assay”, verified if polymer interacts with the host cell and protects it from the infection. Cells were incubated with 100 µL of PSSNa (500 µg/mL) in growth media for 30 min at 37 °C. Then, cells were washed thrice with PBS and infected with 100 µL ZIKV (TCID50 = 400/mL). After four days of infection at 37 °C, supernatants were collected, and the number of ZIKV RNA copies was assessed using RT-qPCR.

Assay III, “Virus attachment assay”, allowed examining if tested polymers block the attachment of virus particles to the host cell. Cells were pre-cooled at 4 °C, overlaid with 100 µL of PSSNa (500 µg/mL) and ZIKV (TCID50 = 400/mL). Cells were incubated at 4 °C to allow the virus attachment, but not internalization to the host cell. Next, cells were washed thrice with PBS, and 100 µL of fresh media was added. Cells were incubated for four days at 37 °C, and supernatants were collected for RT-qPCR analyses.

Assay IV, “Virus replication, assembly, and egress assay”, examined whether compounds hamper late stages of ZIKV replication. Cells were infected with 100 µL ZIKV (TCID50 = 400/mL) and incubated for 2 h at 37 °C to allow the virus to enter the cells. After incubation, cells were rinsed thrice with PBS, and 100 µL of PSSNa polymers in the growth medium was applied. Cells were incubated for four days at 37 °C, and supernatants were collected for RT-qPCR analyses.

### 2.6. RNA Digestion Assay

ZIKV stock (100 μL) was mixed with 1 mg/mL PSSNa or H_2_O at room temperature for 1 h. Then, RNase (A&A Biotechnology, Gdynia, Poland) was added to the final concentration of 100 µg/mL and incubated for 1 h at 37 °C. We used 1% Triton X-100 (Sigma–Aldrich, Poznan, Poland) as a positive control. Then, RNA isolation and RT-qPCR analyses were performed as described above. To ensure that a high concentration of PSSNa does not affect RNA isolation and analyses, PSSNa control samples without RNase were included.

### 2.7. Confocal Microscopy

Cells were seeded on coverslips in 12-well plates (TPP, Trasadingen, Switzerland). After infection, cells were fixed using 4% formaldehyde, permeabilized with 0.1 Triton X-100, and non-specific binding sites were blocked by incubation with 5% bovine serum albumin (BSA; Bioshop Burlington, ON, Canada) in PBS for 2 h. Subsequently, cells were incubated for 2 h with 1 μg/mL rabbit anti-ZIKV envelope antibody (GeneTex, Irvine, CA, USA). After three PBS washes, cells were incubated for 1 h with 2.5 µg/mL of Atto 488 goat anti-rabbit antibody and phalloidin conjugated with Alexa Fluor 647 (0.2 U/mL, Invitrogen, Warsaw, Poland). Subsequently, cells were washed thrice with PBS and nuclei (DNA) were stained with DAPI (0.1 μg/mL, Sigma–Aldrich, Poznan, Poland) for 20 min at room temperature. Cells were washed with PBS and coverslips with immunostained cells were mounted on glass slides in ProLong Diamond Antifade Mountant medium (Life technologies, Eugene, OR, USA) and sealed. Fluorescent images were acquired using Zeiss LSM 710 confocal microscope (Carl Zeiss Microscopy GmbH, Jena, Germany) and processed using ImageJ (ver. 1.52b) Fiji software (Madison, WI, USA) [38]. The number of viral particles and the number of cells was calculated using ImageJ “3D Objects Counter” tool with parameters determined by comparison to mock-treated cells [39]. Obtained data were checked for normality using Shapiro–Wilk test, and one-way ANOVA with Dunnet test for multiple comparisons was carried out. A *p*-value < 0.05 was considered significant (*).

### 2.8. Surface Binding Assay

The assay was performed as described before with some modifications. To test whether virions bind to PSSNa, coverslips were placed in 12-well plates, overlaid with 40 μg/mL bovine type I collagen (PureCol, Advanced BioMatrix, US) in PBS and incubated overnight at 37 °C. Then, coverslips were washed twice with PBS, and tested compound or control (PBS) were added. Coverslips were incubated at 37 °C for 2 h and washed with PBS. Subsequently, a virus or mock samples were applied. Samples were incubated at 37 °C for 2 h. Then, coverslips were washed twice with PBS, fixed with 4% paraformaldehyde, permeabilized, and immunostained as described in confocal microscopy section. The number of virions for each focal plane was assessed using the ImageJ Fiji software (National Institutes of Health, Bethesda, MD, USA).

### 2.9. Statistics

All experiments were conducted at least three times. The results are expressed as means ± standard deviations (SD). Half-maximal inhibitory concentration (IC50) and half-maximal toxic concentration (TC50) values were calculated using the Graph Pad Prism 7.0 function. To determine the significance of the results, Student *t*-test was used, and *p* < 0.05 was considered significant.

## 3. Results

### 3.1. PSSNa Shows No Toxicity Towards Somatic, Glioblastoma and Primary Cells

**Figure 1:**
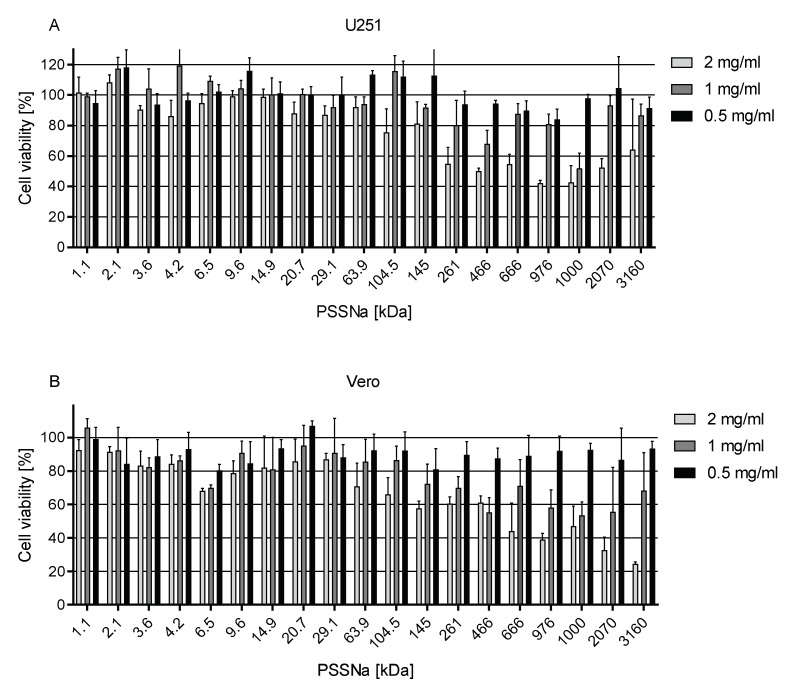
Cytotoxicity of PSSNa of various MWs at 2000, 1000, and 500 µg/mL. Results of XTT assay of the tested polymers on U251 (**A**) and Vero cells (**B**). All experiments were performed in triplicate. Average values with standard deviations (error bars) are presented.

XTT assay was carried out to assess the cytotoxicity of PSSNa. Confluent cells were incubated with solutions of PSSNa of different molecular weights (MW; 19 compounds) at various concentrations for three (Vero cells), four (U251 cells), or five days (HSF cells), reflecting the ZIKV replication time for each cell type. Our results indicate moderate cytotoxicity towards all tested cell lines only at very high doses of PSSNa (>2 mg/mL) of high MW (Figure 1 and Figure S1A). For the intermediate dose of 1 mg/mL the cytotoxicity could not be monotonically correlated to their MW, but PSSNa of >105 kDa were amongst the most toxic ones. For PSSNa of lower MW we did not observe any marked cytotoxicity at any concentration.

### 3.2. PSSNa Exhibit Strong Anti-ZIKV Properties

**Figure 2:**
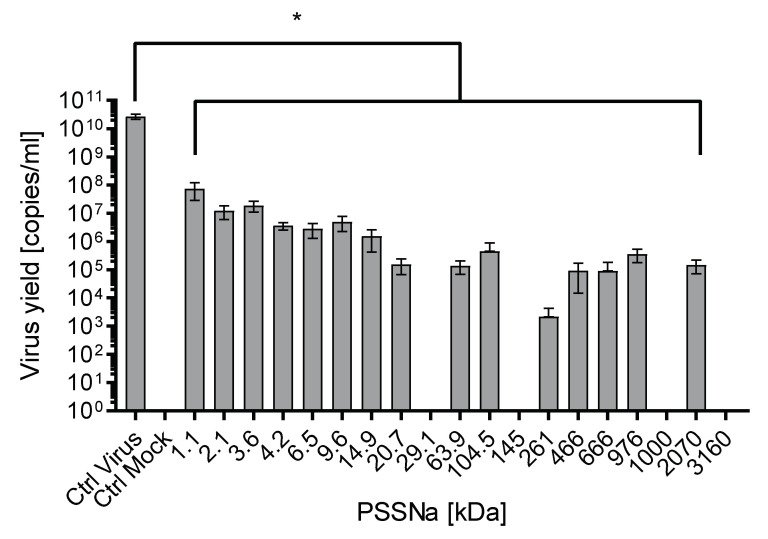
PSSNa inhibition of ZIKV replication correlates with the MW of the polymer. The assay was carried out in the U251 cells, infected with the ZIKV H/PF/2013 virus in the presence of different polymers at the concentration of 0.5 mg/mL. Inhibition of the infection was evaluated using RT-qPCR. Data are shown as virus yield (copies of viral genome per milliliter). All experiments were performed in triplicate. The results are presented as average values with standard deviations (error bars). An asterisk (*p* < 0.05) indicates values that are significantly different from the control.

In Assay I, U251 cells was infected with ZIKV in the presence or absence of each of 19 tested PSSNa polymers of different MW. RT-qPCR analyses showed potent inhibition of ZIKV replication at 0.5 mg/mL. Also, results indicate that in many cases PSSNa at this concentration limited ZIKV replication below the RT-qPCR detection threshold (1000 RNA copies per mL). We also observed that the inhibitory activity correlates, to some extent, with the MW of the polymer. As indicated in Figure 2, PSSNa polymers of higher MW exhibit a more substantial inhibitory effect (>4 logs inhibition), while polymers of lower MW showed weaker inhibition (2–4 logs inhibition). However, this correlation had to be tested later using lower PSSNa concentrations. Therefore, for further studies, four polymers with MWs covering a broad range (1.1, 20.7, 29.1, and 3160 kDa) were selected.

**Figure 3:**
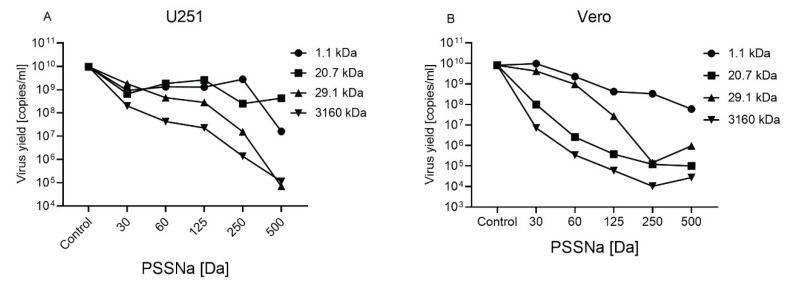
The inhibition of ZIKV H/PF/2013 replication cycle by PSSNa in different cell types. Inhibition of the infection was evaluated using RT-qPCR. U251 (**A**) and Vero (**B**) cells were infected in the presence of appropriate PSSNa concentrations for three and four days, respectively. Data are shown as logarithmic values of ZIKV RNA copy number per milliliter with standard deviation (error bars). The results are presented as average values of at least three replications.

In addition to U251 cell line, virus inhibition assay was also carried out in Vero and HSF cells to ensure that the antiviral effect is not cell-dependent. Experiments showed a similar pattern of inhibition in Vero and U251 cells (Figure 3). Again, a positive correlation between MW of particular PSSNa and the inhibition efficacy was observed. However, in the case of U251 cells a dose-dependent virus inhibition was noted only for PSSNa 29.1 and 3160 kDa, with IC50 of 14.3 and 8.8 µg/mL, respectively. In Vero cells, all tested PSSNa polymers hampered ZIKV infection in a dose-dependent manner. Low cytotoxicity and strong inhibition of ZIKA replication is expressed as a selectivity index (SI). The calculated SI values were high for U251 and Vero cells and are listed in Table 1. Moreover, in HSF cells, complete inhibition (below RT-qPCR detection limit) of ZIKV replication was observed in the case of all tested PSSNa polymers (1.1, 20.7, 29.1, and 3160 kDa) at 250 µg/mL (Figure S1B).

**Figure 4:**
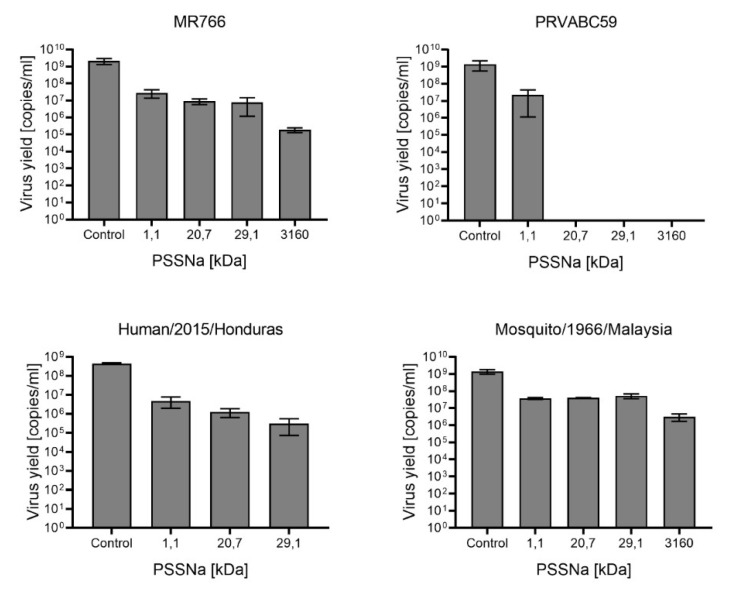
Inhibition of different strains of ZIKV replication cycle by PSSNa. U251 cells were infected with the virus in the presence of different polymers for four days. Inhibition of the infection was evaluated using RT-qPCR. Data are shown as virus yield (copies of viral genome per milliliter). The results are presented as average values of at least three replications.

We also study whether the observed inhibitory effect of PSSNa would be observed in the case of other ZIKV strains. Cells were infected with four ZIKV strains of both Asian and African lineage and isolated from different host species (MR 766 (Rhesus/1947/Uganda), PRAVABC59 (Human/2015/Puerto Rico), Human/2015/Honduras, Mosquito/1966/Malaysia) in the presence of 250 µg/mL PSSNa. Obtained results indicate that PSSNa strongly suppresses the replication of all tested ZIKV strains. As before, it was observed that the PSSNa inhibitory properties depend on the length of the polymer chain. The strongest effect was observed against the human Asian PRAVABC59 strain, where all tested polymers except for 1.1 kDa inhibited the ZIKV replication below the detection limit of RT-qPCR (Figure 4). PSSNa hampers the replication of African lineage strain MR766. The molecular weight correlated with the PSSNa inhibitory activity. While polymers with shorter chaining decreased the virus yield 100 times, for PSSNa of 3160 kDa 1000 reduction in virus yield was recorded. Weaker inhibition was observed for strains Human/2015/Honduras and Mosquito/1966/Malaysia strains. The differences observed may be due to variability within the E protein between the strains. However, advanced comparative genetic studies are needed to test that hypothesis.

### 3.3. PSSNa Interacts Directly with ZIKV Virions, Preventing their Attachment to the Host Cell

**Figure 5:**
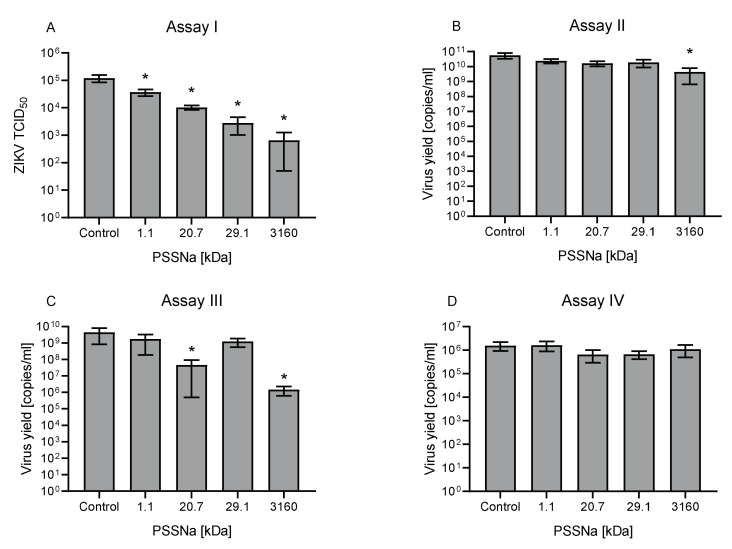
PSSNa interacts with ZIKV particles and blocks their interaction with the receptor. Inhibitory activity of PSSNa of different MW at different stages of ZIKV infection cycle. Functional Assays I–IV were carried out on U251 cells as described in the Materials and Methods section using H/PF/2013 strain after the virus inactivation Assay I (Assay I, (**A**)), where the virus was pre-incubated with polymers, diluted and titrated on U251 cells. Modulation of virus yield as determined by RT-qPCR after the Cell protection assay (Assay II, (**B**)); Attachment assay (Assay III, (**C**)); or Virus replication, assembly, and egress assay (Assay IV, (**D**)). The inhibition of the infection was evaluated using RT-qPCR. All experiments were performed in triplicate. The results are presented as average values with standard deviations (error bars). An asterisk (*p* < 0.05) indicates values that are significantly different from the control.

To further explore the mechanism of action, four functional Assays (I-IV) were performed on U251 cells. The results suggest that PSSNa inactivated ZIKV particles and prevented their interaction with the host cells. Assay I showed that the preincubation of the virus with PSSNa restricted ZIKV viability (Figure 5). Also, here a correlation between the MW of the polymer and its antiviral activity was obvious. Some inhibition was also observed in other assays where the polymer is present during the early stages of viral infection, but the effect was non-significant or mild (Assays II and III). While one cannot exclude an additional secondary mechanism of action, we believe that the observed effect also results from the virus–polymer interaction. The polymers did not inhibit virus replication as shown in Assay IV, where polymers were incubated with cells after the infection. In order to avoid multiple cycles of infection, assay IV was carried out using ZIKV at TCID50 of 10,000 per mL, and cells were incubated for 12 h, which corresponds to the reported Flaviviridae replication cycle [40]. Consequently, the functional assays showed that the PSSNa prevents virus attachment to the host cell, and in such a way, it hampers virus replication.

**Figure 6:**
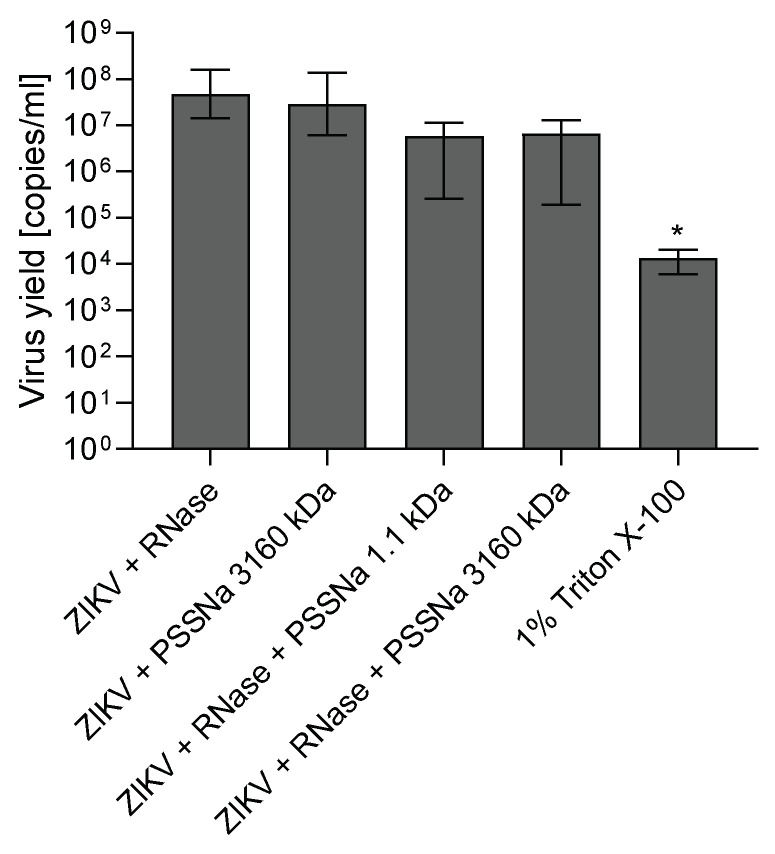
PSSNa does not disrupt ZIKV particle integrity. ZIKV H/PF/2013 stock was incubated with 1 mg/mL PSSNa, then released RNA was digested with 100 µg/mL RNase. 1% Triton X-100 was used as a positive control. Results show RT-qPCR analyses (copies of viral genome per milliliter). The results are presented as average values with standard deviation (error bars) of at least three replications. An asterisk *(p* < 0.05) indicates values that are significantly different from the control.

To further study the mechanism of action of PSSNa, an RNA digest assay was performed to study whether the interaction between ZIKV and PSSNa disrupts virion integrity, resulting in the release of the vRNA from the virus particle. The obtained results indicate only minor, non-significant drop in ZIKV RNA copies in PSSNa samples compare to Triton X-100 control, suggesting that PSSNa doesn’t disrupt ZIKV particle integrity (Figure 6).

**Figure 7:**
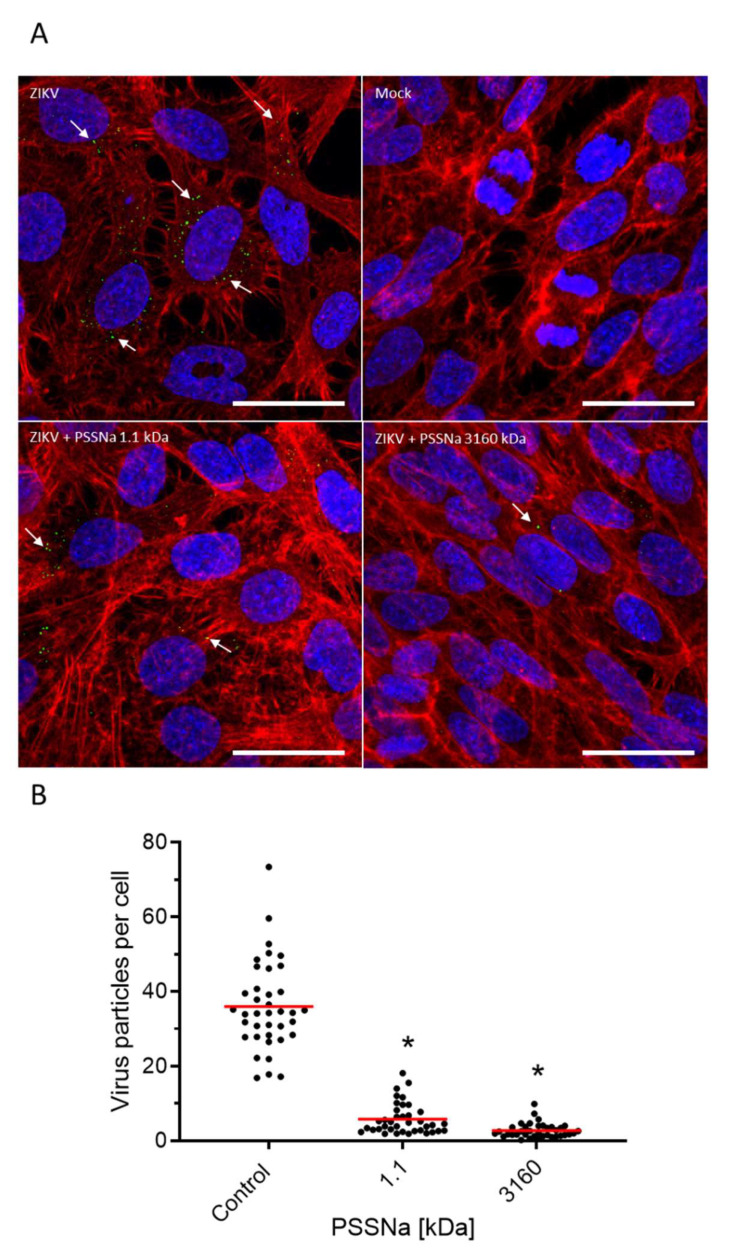
PSSNa blocks the attachment of ZIKV to host cells. Vero cells were inoculated with ZIKV H/PF/2013 (TCID50 = 10,000/mL) in the presence of 250 µg/mL PSSNa of 1.1 or 3160 kDa or control buffer. Cells were incubated at 4 °C for 2 h, fixed, and confocal images were collected. (**A**) Cell nuclei are denoted in blue, β-actin in red, and ZIKV E protein is denoted in green (indicated by arrows). Scale bar = 100 µm. (**B**) Confocal microscopy analysis of the number of particles on the surface of cells treated with PSSNa. The number of particles and the number of cells was assessed using ImageJ Fiji 3D Objects Counter tool. Each set shows data from a minimum of 45 fields of view registered from 7 different samples prepared in three independent experiments. An asterisk (*p* < 0.05) indicates values that are significantly different from the control.

The E protein dimer displayed on the surface of mature ZIKV virions is responsible for the host cell entry factor binding and fusion [40]. Confocal images confirmed that PSSNa prevents the virus attachment to the cell. At low temperature blocking the virus internalization, PSSNa decreases the number of viral particles attached to the cell (Figure 7). Thus, the presented results suggest a direct interaction of PSSNa with E protein, which in turn hinders virus entry into the host cells and prevents virus replication.

## 4. Discussion

Since the epidemic outbreak on Yap Island in 2007 and subsequent epidemics in Polynesia and Brazil, ZIKV remains one of the most important health concerns worldwide. Unfortunately, despite the extensive efforts of researchers, doctors, and epidemiologists, no specific therapy or prophylactic tools have been developed. While health concerns related to the emergence of the novel coronavirus arise, one should remember that flaviviruses have already shown their potential, and the story of Zika may not be over yet.

Natural and synthetic polymers play a crucial role in pharmaceutical research and the development of new drugs. Currently, polymers are most often used as inactive carriers of low molecular weight drugs, increasing their bioavailability and stability. However, increasing evidence suggests their possible use as drugs. Unlike low molecular weight drugs, polymers exhibit multivalency of interactions with their molecular target, often mimicking natural processes such as attachment of viruses to the surface of a host cell. This is a mechanism absent in the case of low molecular weight drugs that can bring major therapeutic benefits. Despite the skeptical approach to the results of research conducted since the 1960s, new methods of design and synthesis, characterization of polymers enable the production of polymers with precisely defined biological functions and properties. For this reason, new synthetic polymers are likely to be used as drugs in many areas of medicine, including antiviral drugs.

In this work, we have shown that a negatively charged polymer, poly(sodium 4-styrenesulfonate) (PSSNa) can efficiently hamper ZIKV infection in vitro by directly binding to the virus and preventing the attachment of the virus to the host cell. PSSNa vastly decreases the viral replication in vitro in a number of cellular models, indicating broad specificity regardless of the model. The low toxicity of this synthetic polymer is also advantageous. Importantly, PSSNa can effectively treat ongoing infections, without loss in antiviral activity over time, as indicated in Assay IV. Importantly, we showed that PSSNa inhibits different ZIKV strains derived from various host species including humans, rhesus monkey, and mosquito. Further studies, however, are required to find out if PSSNa can permanently inactivate ZIKV virions or produce a stable virus–PSSNa complex. Therefore, another possibility is to utilize PSSNa as an “active antiviral platform” for low molecular weight drugs, peptides or neutralizing antibodies. Such combination may improve the effect of low molecular weight inhibitors, reduce their toxicity, or reduce their effective virus inhibitory doses.

Analysis of the potential binding sites on the viral particle suggests that PSSNa may bind with the fusion loop of E protein dimer, as it has been shown that the ZIKV fusion loop of E protein dimer is positively charged and is likely to interact with anionic compounds [41]. In all assays, the superiority of high molecular weight PSSNa compared to the ones with lower MW was observed. One may speculate that such an activity may result from the interaction between the polymer and several concatamerically distributed E protein molecules on the virus sphere. In such a mechanism, a single, long, flexible polymer chain simultaneously blocks many potential sites of virus protein responsible for binding to cell attachment factors or entry receptors. Furthermore, similarly, as we described earlier for the HTCC molecule antiviral activity directed against corona viruses we speculate that the “mechanism of the zipper”, where the sum of weak interactions results in effective and selective inhibition [14,42].

In summary, for the first time, we presented a systematic study on the inhibitory action of PSSNa on the ZIKV entry. Poly(sodium 4-styrenesulfonate) is an already FDA approved drug (Kayexalate) for treating hyperkalemia as a potassium binder administered orally. Tolevamer, a soluble anionic poly(sodium 4-styrenesulfonate), was developed for the treatment of Clostridium difficile associated diarrhea. It did not pass Phase III clinical trials due to poor response to therapy (47%) [43]. These examples confirm the therapeutic potential of the PSS salts. Meticulous in vivo and clinical studies will allow exploring the potential of PSSNa in the treatment of Zika virus infections. Our study proves that high activity and low toxicity, as well as simple chemical structure and an array of possible modifications indicate the high potential of PSSNa as an anti-ZIKV agent.

## Figures and Tables

**Table 1:** Inhibition of ZIKV H/PF/2013 virus in various cellular models. Experimental values of half-maximal inhibitory concentration (IC50), half-maximal toxic concentration (TC50), and selectivity index (SI) of PSSNa for ZIKV in different cell lines. The “-” indicates that the given values cannot be determined.

**U251 Cells**				
	**1.1 kDa**	**20.7 kDa**	**29.1 kDa**	**3160 kDa**
IC50 (µg/mL)	9.0	8.1	14.4	8.8
TC50 (µg/mL)	-	2335.0	8495.6	2938.3
SI	-	288.3	590.0	333.9
**Vero Cells**				
	**1.1 kDa**	**20.7 kDa**	**29.1 kDa**	**3160 kDa**
IC50 (µg/mL)	53.5	13.1	30.9	8.4
TC50 (µg/mL)	2530.8	4345.5	-	1828.0
SI	47.3	331.0	-	217.6

## Supplementary Materials

The following are available online at https://www.mdpi.com/1999-4915/12/9/926/s1, Figure S1: title, Table S1: PSSNa inhibition of ZIKV replication in Human Skin Fibroblasts (HSF).
