# Supplementary material for: In Vitro Inhibition of Zika Virus Replication with Poly(Sodium 4-Styrenesulfonate)
Source: Viruses. 2020 Aug 23;12(9):926. doi: 10.3390/v12090926 (PMC7551931; doi:10.3390/v12090926)
Supplement: Supplementary file 1 [file viruses-12-00926-s001.pdf]

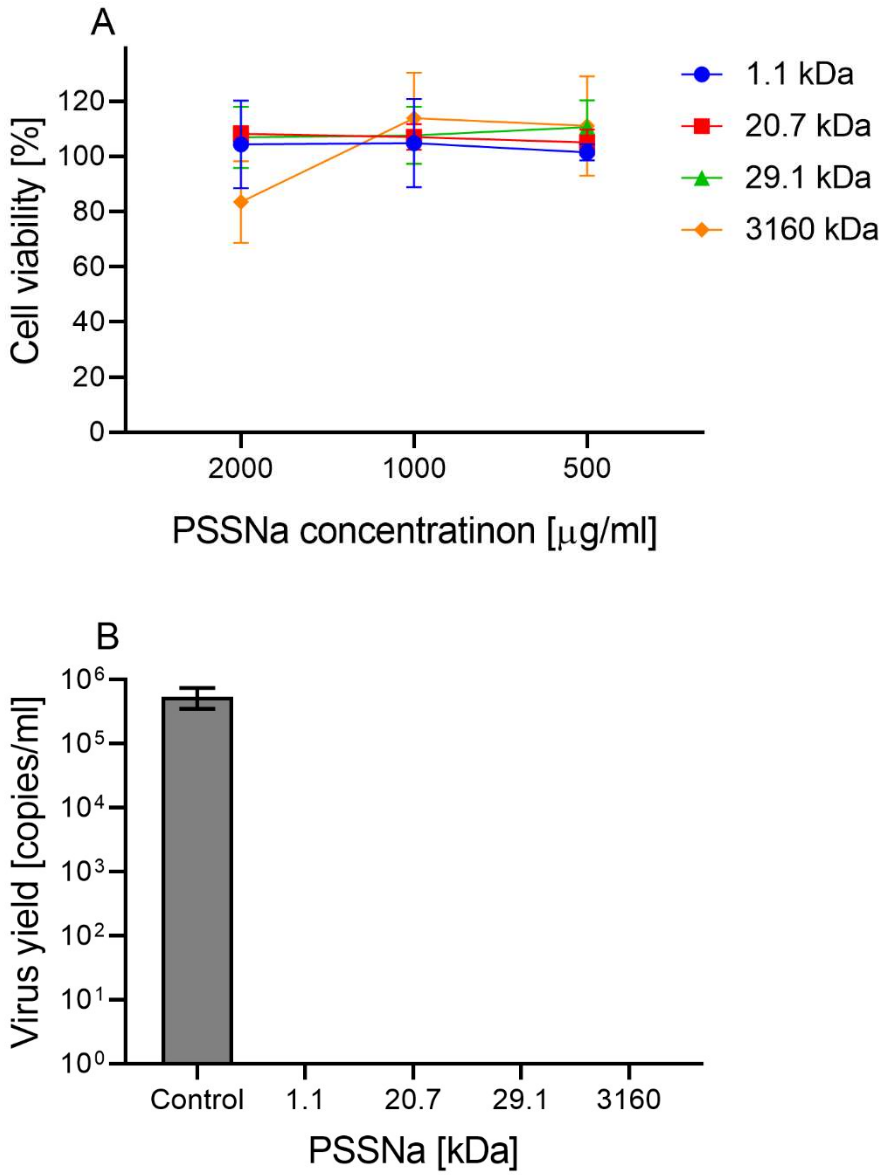

Figure S1. PSSNa inhibition of ZIKV replication in Human Skin Fibroblasts (HSF). (A) Results of XTT assay of the tested polymers on HSF cells. (B) HSF cells infected with the virus in the presence of PSSNa of different Mw at the concentration of 250 μg/ml. Inhibition of the infection was evaluated using RT qPCR. Data are shown as virus yield (copies of viral genome per milliliter). All experiments were performed in triplicate. The results are presented as average values with standard deviations (error bars).
